# Basophils response to Pru p 3 and Ara h 9 in patients sensitised to peach under specific immunotherapy

**DOI:** 10.1186/2045-7022-4-S2-O19

**Published:** 2014-03-17

**Authors:** Francisca Gomez, Enrique Gomez, Inmaculada Doña, Luisa Galindo, Paloma Campo, Teresa Posadas, Maria Jose Torres, Araceli Diaz-Perales, Miguel Blanca, Lina Mayorga

**Affiliations:** 1 Hospital Carlos Haya, Malaga, C/Canillas de Aceituno Nº 5 Portal 2 4º D, Málaga, 29004, Spain; 2 Research Laboratory, Carlos Haya Hospital-FIMABIS, Málaga, Spain; 3 Allergy Service, Carlos Haya Hospital, Málaga, Spain; 4 Plant Biotechnology Institute (UPM-INIA), Madrid, Spain

## Rationale

In Southern Europe Pru p3 is the primary sensitizer of plants fruit and it is responsible of severe reactions. Specific immunotherapy (SIT) brings a new perspective to treat those patients. There is a lack of knowledge regarding cellular responses that include changes in the basophil activation during the IT. We aim to analyse early changes in the basophil response to Pru p 3 and other related allergen (Ara h 9) after the first month of sublingual immunotherapy (SLIT).

## Methods

Forty-six peach allergic patients confirmed by positive specific IgE determined by skin prick test or fresh peach (prick-by-prick), ImmunoCAP IgE and/or a double blind placebo control food challenge with peach. Basophil reactivity was determined by the basophil activation test (BAT) with Pru p 3 and Ara h 9 at two concentrations, 1 and 0.1 µg/ml, before and after 1month of SLIT.

## Results

Twenty one patients evaluated (45%) performed anaphylaxis and 25 (55%) urticaria and/or angioedema. The 82,6% showed sensitization to other plant foods proteins and 69,5% showed sensitization to pollens. The BAT was done in 25 patients with first month of SLIT completed. The 28% patients have an increase of Pru p 3 reactivity. The 36% patients showed same reactivity after first month and 36% presented a decreased reactivity to Pru p3. Similar results were obtained for Ara h 9 in those patients.

## Conclusion

Preliminary result disclosed that percentage of patients who underwent changes in BAT reactivity to Pru p3 and Ara h 9 was similar. There have been no differential clinical pattern in the groups studied after one month of SLIT. The BAT shows a good correlation between both Pru p 3 and Ara h 9.

